# L-Arginine Supplementation in Type II Diabetic Rats Preserves Renal Function and Improves Insulin Sensitivity by Altering the Nitric Oxide Pathway

**DOI:** 10.1155/2014/171546

**Published:** 2014-01-12

**Authors:** Taylor Claybaugh, Sarah Decker, Kelly McCall, Yuriy Slyvka, Jerrod Steimle, Aaron Wood, Megan Schaefer, Jean Thuma, Sharon Inman

**Affiliations:** ^1^Department of Biomedical Sciences, Heritage College of Osteopathic Medicine, Ohio University, 228 Irvine, Athens, OH 45701, USA; ^2^The Diabetes Institute at Ohio University, Ohio University, 228 Irvine, Athens, OH 45701, USA; ^3^Department of Specialty Medicine, Heritage College of Osteopathic Medicine, Ohio University, 228 Irvine, Athens, OH 45701, USA

## Abstract

Rat studies demonstrated that type II diabetes mellitus (T2DM) decreases both the production and bioavailability of nitric oxide (NO). L-arginine (LA) provides the precursor for the production of NO. We hypothesized that LA dietary supplementation will preserve NO production via endothelial nitric oxide synthase (eNOS) causing renal microvascular vasodilation and increased glomerular blood flow and thus increasing glomerular filtration rate (GFR). This would impede the formation of reactive oxygen species which contributes to cell damage and death. LA supplementation preserved GFR in the treated diabetic rats compared to untreated diabetic rats. We provide evidence that this effect may be due to increased levels of eNOS and urinary cyclic guanosine monophosphate, which leads to renal microvascular vasodilation. Plasma nitrotyrosine was decreased in the LA treated rats; however, plasma nitrite levels remained unaffected as expected. Marked improvements in glucose tolerance were also observed in the LA treated diabetic rats. These results demonstrate that LA supplementation preserves NO activity and may delay the onset of insulin resistance and renal dysfunction during hyperglycemic stress. These results suggest the importance of the NO pathway in consequent renal dysfunction and in the development of insulin resistance in diabetic rats.

## 1. Introduction

Diabetic nephropathy is the number one cause of end-stage renal failure [1]. The pathogenesis of diabetic nephropathy is thought to occur in two major stages. First, there is a vasodilation in the pre- and postglomerular arterioles, which leads to higher intraglomerular blood flow and pressure. This causes an early hyperfiltration in the nephrons and eventually damages the glomerular membrane and allows for proteins, glucose, and other molecules to be filtered by the glomerulus and then excreted in the urine. Secondly, the final irreversible stage is a vasoconstriction of the glomerular arterioles, which results in low blood flow and glomerular filtration rates [2].

**Figure 1:**
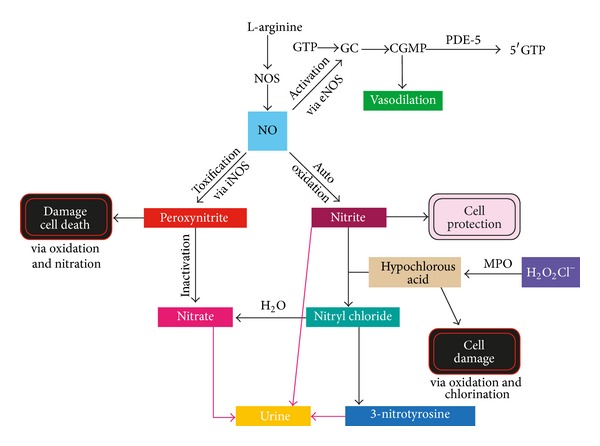
Nitric oxide biosynthetic pathway.

The nitric oxide (NO) system has been shown to be altered in diabetes and in diabetic nephropathy [3]. Nitric oxide is a vasodilator and if it is deficient or its metabolism is altered, this affects renal function and insulin sensitivity [4]. The precursor for NO is L-arginine (LA). LA is an amino acid that is synthesized within the body and can also be found in various types of food. LA is converted to NO by nitric oxide synthase (NOS) [5]. Endothelial NOS (eNOS) results in NO release from the endothelium of blood vessels and causes vasodilation via cyclic guanosine monophosphate (cGMP) [6]. Inducible NOS (iNOS) is an isozyme that is present in an oxidative environment. High levels of iNOS produce larger amounts of NO, which allows NO to react with superoxide forming peroxynitrite and thus leads to cell toxicity and/or death [7]. Therefore, higher levels of renal eNOS compared to iNOS would be beneficial in the late stages of diabetic nephropathy to maintain renal blood flow via vasodilation. A schematic of the NO biosynthetic pathway is provided in Figure 1.

While it is true in the early stages of diabetic nephropathy that there is renal vasodilation and hyperfiltration, it is in the later stages that glomerular filtration rate decreases and the continued availability of nitric oxide would maintain glomerular filtration rate and renal blood flow [8]. Our hypothesis is that nitric oxide bioavailability may be increased by L-arginine treatment in the later stages of diabetic nephropathy. It is upregulated by endothelial nitric oxide synthase rather than by the arginase enzyme.

LA deficiency causes endothelial inflammation and cardiovascular disorders, and dietary LA supplementation can reverse these disorders [9, 10]. In patients with type 2 diabetes mellitus (T2DM), LA supplementation resulted in a significant increase in NO concentration and total antioxidant status of the patient [11]. In a rat model of T2DM, the obese Zucker rat, it has been reported these rats had significantly lower renal function compared to the diabetic animals fed an antioxidant diet. This suggests that in an oxidative stress environment renal function declines and may be due to the increased production of peroxynitrite via iNOS resulting in cell death [12].

Therefore, we hypothesized that LA dietary supplementation will alter the NO biosynthetic pathway and preserve renal function in diabetic Wistar rats. To test this hypothesis, we utilized the Wistar rat model of T2DM [13] and evaluated the effects of LA dietary supplementation on four components of the NO pathway: renal eNOS and iNOS protein levels, urinary cGMP and plasma nitrotyrosine, and nitrite. We report the novel findings that LA supplementation preserves glomerular filtration rate via alterations in the NO pathway and improves insulin resistance in diabetic Wistar rats.

## 2. Materials and Methods

### 2.1. Type 2 Diabetic Rat Model and Groups

All of the animal work was conducted with approval from the Ohio University Institutional Animal Care and Use Committee.

Male Wistar rats, 27 in total, were purchased from Harlan Laboratories (Indianapolis, IN) at 6 weeks of age. After being acclimated to laboratory conditions for 4 days, they were randomly divided into three experimental groups (*n* = 9 rats/group). Group 1 served as the nondiabetic controls (Nondiabetic Control) and was fed a standard ad libitum diet (Purina Mills, Inc., St. Louis, MO, number 5012). Group 2 served as the type 2 diabetic controls (Diabetic Control) and was fed a high (61%) sucrose (HS) diet (Purina Mills Inc., St. Louis, MO, number 58R1). Group 3 (LA) was fed the same HS diet with additional 1 g/kg body weight L-arginine supplementation given twice daily via oral gavage (Sigma-Aldrich Corporation, St. Louis, MO, number A5131). Each group was maintained on their respective diet for a total of 8 weeks.

### 2.2. Testing Protocol

Before being placed on the experimental diets, rat body weights were recorded and then taken weekly. Fasting blood glucose measurements were taken weekly using a One Touch Glucometer (Johnson and Johnson). At the initiation of the diet, each rat was placed in a metabolic cage with access to water solely for a 24-hour urine collection. Glomerular filtration rate (GFR) based on creatinine clearance was determined using urine and plasma creatinine assay kits (Cayman Chemicals, Ann Arbor, MI, number 500701 and number 700460) and urine output levels. GFR was calculated by urine concentration multiplied by the urine output, all divided by plasma concentration. cGMP urine levels were measured using a kit from Cayman Chemicals (Cayman Chemicals, Ann Arbor, MI, number 581021). Plasma nitrotyrosine measurements were made using a nitrotyrosine assay kit (Hycult Laboratories, Uden, the Netherlands, number HK501). These measurements were repeated at experimental times of 3, 6, and 8 weeks. A glucose tolerance test (GTT) was performed at week 6. Blood glucose readings for the GTT were taken at 0, 15, 30, 60, 90, 120, and 150 minutes after intraperitoneal injection of glucose. Plasma nitrite levels were measured using a chemical assay kit (Cayman Chemicals, number 780001) and were tested at weeks 0 and 8.

### 2.3. Posteuthanasia Analysis

At the end of the 8-week period, rats were euthanized and one kidney was removed; the renal medulla and renal cortex were separated and collected and stored in liquid nitrogen for renal eNOS and iNOS protein level analyses by Western blot.

### 2.4. Western Blot Analyses of eNOS and iNOS

Western blots were performed to assess the renal cortex and medulla protein levels of eNOS and iNOS. Short isoform-specific primary antibodies to eNOS (1 : 1000 dilution, SC-654, Santa Cruz Biotech, Santa Cruz, CA) and iNOS (iNOS-A, 1 : 2000, Alpha Diagnostics International, San Antonio, TX) were used. Goat anti-rabbit horseradish peroxidase-conjugated secondary antibody (1 : 2000 dilution, number 20320, Alpha Diagnostics International, San Antonio, TX) was subsequently applied. *β*-Actin was measured as an internal control using a monoclonal primary antibody (1 : 2000 dilution, A2228, Sigma-Aldrich Corporation, St. Louis, MO) and horseradish peroxidase-conjugated goat anti-mouse secondary antibody (1 : 30 000 dilution, A9044, Sigma-Aldrich Corporation, St. Louis, MO). Membranes were detected with ECL immunoblotting detection reagents (GE Healthcare, Piscataway, NJ) and bands were quantified using a Chemi Doc chemiluminiscent detection system and Quantity One software (Bio-Rad, Richmond CA). The results were expressed as NOS/*β*-actin density ratio.

### 2.5. Statistical Analysis

As the main analytic framework multilevel modeling was applied to all dependent variables. Each model contained one between-subjects factor treatment (TX: Control, Diabetic Control, L-arginine) and one within-subjects factor time. In each model the baseline level was included as a covariate to increase power to detect group differences. At each time point pairwise comparisons between all possible pairs of groups were performed. The significance level was set to 0.05. In the figures data are presented as means ± standard errors.

## 3. Results 

### 3.1. Weight Gain

**Figure 2:**
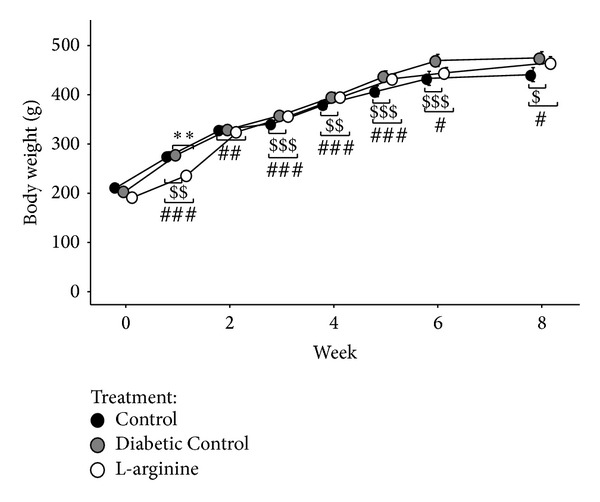
Effect of the high fructose diet alone (Diabetic Control) and high fructose plus L-arginine diet (L-Arginine) on weight again over 8 weeks. Data are represented as means ± standard deviations. Control versus Diabetic Control: ^$^ *P* < 0.05, ^$$^ *P* < 0.01, and ^$$$^ *P* < 0.001; Control versus L-arginine: ^#^ *P* < 0.05 and ^###^ *P* < 0.001; Diabetic Control versus L-arginine: **P < 0.01.

The three groups of rats gained weight at different rates. The LA rats were heavier than the Nondiabetic Control rats at all weeks except weeks 0, 1, and 2 but were lighter than the Diabetic Control rats at all weeks (Figure 2). Similarly, the Diabetic Control rats were heavier than the Nondiabetic Control rats at all weeks except for weeks 0 and 2 (Figure 2).

### 3.2. Fasting Blood Glucose

**Figure 3:**
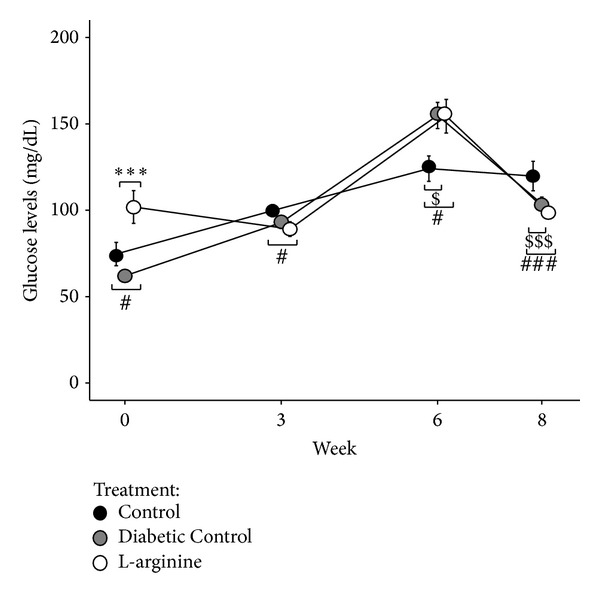
Effect of the high fructose diet alone and high fructose plus L-arginine diet on fasting glucose levels over 8 weeks. Control versus Diabetic Control: ^$^ *P* < 0.05 and^$$$^ *P* < 0.001; Control versus L-arginine: ^#^ *P* < 0.05 and ^###^ *P* < 0.001; Diabetic Control versus L-arginine: ***P < 0.001.

Glucose levels in LA rats were higher at weeks 0 and 6 but lower at weeks 3 and 8 than those in the Nondiabetic Control rats and higher than in the Diabetic Control rats at week 0 (Figure 3). Fasting blood glucose levels in the Diabetic Control rats were higher at week 6 and lower at week 8 than those in the Nondiabetic Control rats (Figure 3). LA supplementation did not improve fasting blood glucose levels at the termination of the experimental protocol time period (Figure 3).

### 3.3. GFR

**Figure 4:**
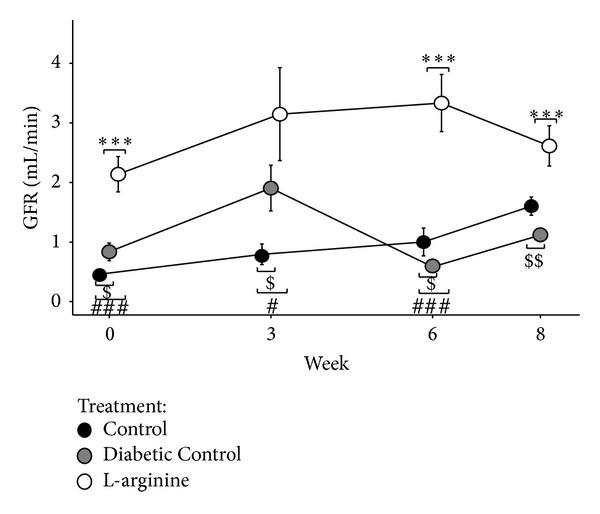
Effect of the high fructose diet alone and high fructose plus L-arginine diet on GFR over 8 weeks. Control versus Diabetic Control: ^$^ *P* < 0.05 and ^$$^ *P* < 0.01; Control versus L-arginine: ^#^ *P* < 0.05 and ^###^ *P* < 0.001; Diabetic Control versus L-arginine: ***P < 0.001.

LA supplementation resulted in higher GFR at all time periods compared to the Nondiabetic Control rats and at weeks 3 and 8 compared to Diabetic Controls (Figure 4). GFRs in the Nondiabetic Control rats were lower than those in the Diabetic Control rats at weeks 0 and 3 but were higher at weeks 6 and 8 (Figure 4).

### 3.4. cGMP

**Figure 5:**
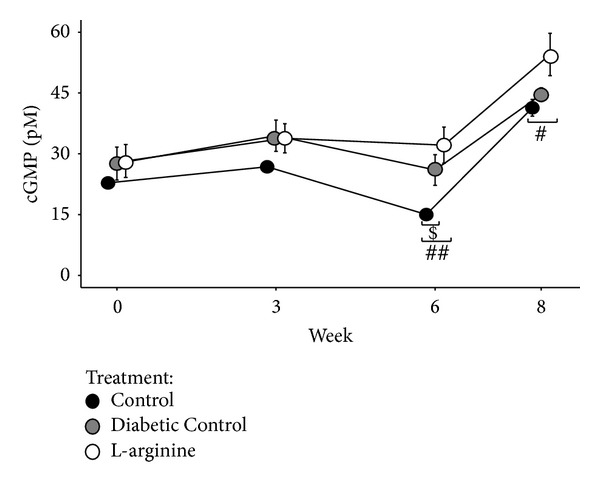
Effect of the high fructose diet alone and high fructose plus L-arginine diet on cGMP levels over 8 weeks. Control versus Diabetic Control: ^$^ *P* < 0.05; Control versus L-arginine: ^#^ *P* < 0.05 and ^##^ *P* < 0.01.

LA supplementation increased cGMP levels in diabetic rats. The Nondiabetic Control rats had significantly lower cGMP levels than the other 2 groups at week 6 and lower than the LA rats at week 8 (Figure 5). It is important to note that the Diabetic Control rats did not have as drastic an increase in cGMP levels as LA and Nondiabetic Control rats at weeks 6 and 8 (Figure 5).

### 3.5. Relative Nitrotyrosine

**Figure 6:**
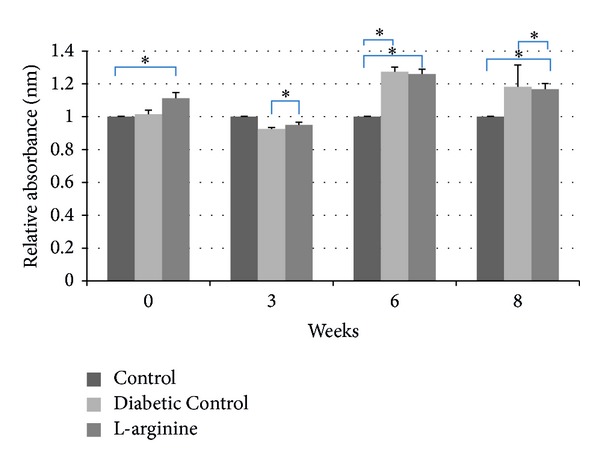
Effect of the high fructose diet alone and high fructose plus L-arginine diet on nitrotyrosine absorbance levels over 8 weeks relative to the Nondiabetic Control. Diabetic Control and L-arginine versus Control: *P < 0.05.

LA supplementation in diabetic rats improved plasma nitrotyrosine levels. LA rats had higher plasma nitrotyrosine levels relative to Nondiabetic Control rats at week 0, but LA rats had higher plasma nitrotyrosine levels relative to Diabetic Control rats at week 3 (Figure 6). At weeks 6 and 8, LA supplemented rats had higher plasma nitrotyrosine levels relative to Nondiabetic Control rats, but lower plasma nitrotyrosine levels relative to Diabetic Control rats (Figure 6). The Diabetic Control rats had higher plasma nitrotyrosine levels relative to both of the other groups at weeks 6 and 8 (Figure 6).

### 3.6. GTT

**Figure 7:**
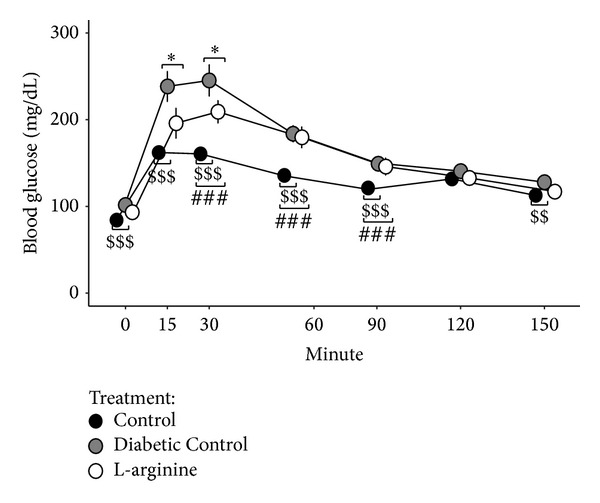
Effect of the high fructose diet alone and high fructose plus L-arginine diet on changes in glucose levels over 150 min in response to a glucose-tolerance test. Control versus Diabetic Control: ^$$^ *P* < 0.01 and ^$$$^ *P* < 0.001; Control versus L-arginine: ^###^ *P* < 0.001; Diabetic Control versus L-arginine: *P < 0.05.

LA supplementation significantly improved glucose tolerance. The Diabetic Control rats had higher glucose levels than the Nondiabetic Control rats at all times after glucose challenge except for 120 minutes, while the LA rats had higher glucose levels than the Nondiabetic Control rats at 30, 60, and 90 minutes (Figure 7). Most importantly, the LA rats had lower glucose levels than the Diabetic Control rats at 15 and 30 minutes after glucose challenge (Figure 7).

### 3.7. Nitrite

**Figure 8:**
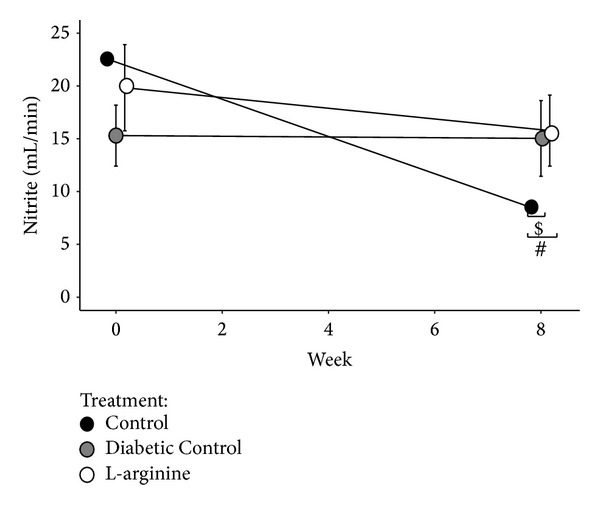
Effect of the high fructose diet alone and high fructose plus L-arginine diet on nitrite over 8 weeks. Control versus Diabetic Control: ^$^ *P* < 0.05; Control versus L-arginine: ^#^ *P* < 0.05.

LA supplementation did not affect nitrite levels compared to Diabetic Control rats (Figure 8). The Nondiabetic Control rats had lower nitrite levels than the other 2 groups at week 8 (Figure 8).

### 3.8. eNOS and iNOS Protein Levels

In the renal cortex and medulla of rats in all groups, eNOS monomers (74, 77, 116, 130, and 150 kDa) and dimers (195, 320, and 380 kDa) and also iNOS monomers (70, 77, 115, 122, and 139 kDa) and dimers (164, 178, 224, 301, and 322 kDa) were detected. The distribution of eNOS and iNOS monomers and dimers by splice forms in the cortex and medulla of different experimental groups is presented in Tables 1 and 2. The eNOS protein levels were higher in both the renal cortex and medulla in LA-treated diabetic rats compared to untreated diabetic rats. Likewise, the iNOS protein levels were lower in both the renal cortex and medulla in LA-treated diabetic rats compared to untreated diabetic rats (Tables 1 and 2).

## 4. Discussion

Dietary L-arginine (LA) supplementation given to type 2 diabetic (T2DM) rats preserved renal function compared to T2DM rats not receiving LA. When LA was supplemented to the diet of T2DM rats, it was converted to nitric oxide (NO) by nitric oxide synthase (NOS). The NO produced was presumably activated via endothelial NOS (eNOS) into guanylyl cyclase (GC). In the biosynthetic pathway, GC is then transformed into cyclic guanosine monophosphate (cGMP) [14]. Due to the rise of NO and eNOS, there were increased levels of the second messenger cGMP that acts on the vascular endothelium causing vasodilation which would increase glomerular filtration rate (GFR). Greater vasodilation slows the progression of renal failure and diabetic nephropathy by maintaining renal blood flow [15]. These findings are important because the results show that LA supplementation may be an inexpensive nutritional treatment for T2DM patients.

It is speculated that renal vasodilation most likely occurred in this study due to the observed greater levels of mediators involved in the NO biosynthetic pathway. GFR, as estimated by creatinine clearance, showed increased filtration for those diabetic rats supplemented with LA. The LA treated diabetic rats did have higher GFRs at the beginning of the study. However, the GFRs of the LA treated diabetic rats remained higher throughout the study compared to untreated diabetic rats. In the pathogenesis of diabetic nephropathy, once GFR is reduced, it is often irreversible [2]. Once the T2DM diagnosis is made, a dietary LA supplementation used as an early intervention may prove to be beneficial in these patients. Increased levels of eNOS and lower levels of iNOS in the renal medulla and cortex were detected in the LA supplemented rats compared to the diabetic control rats. eNOS causes positive activation of NO leading to cGMP and vasodilation [16]. iNOS causes toxification of NO forming peroxynitrite which leads to cell damage and death [17]. Increased urinary cGMP levels in LA supplemented rats were observed. This is most probably due to an overabundance of plasma cGMP being produced from eNOS in the NO pathway.

L-arginine is a substrate for at least 5 enzymes identified in mammals, including arginase, arginine-glycine transaminase, kyotorphin synthase, nitric oxide synthase, and arginine decarboxylase. L-arginine is essential for the synthesis of creatine, urea, polyamines, nitric oxide, and agmatine. Only the utilization via NOS results in a positive effect. A beneficial effect of acute and chronic L-arginine supplementation on endothelial-derived nitric oxide production and endothelial function has been shown in a number of studies. In fact, we saw a beneficial effect on the preservation of glomerular filtration rate in our diabetic model at the conclusion of the study. Therefore, we hypothesize that eNOS was the substrate for L-arginine causing renal microvascular vasodilation and increased renal blood flow and thus glomerular filtration rate. Furthermore, Morris Jr. et al. found that elevated arginase enzyme activity in the diabetic rat kidney inhibits NOS activity and NOS becomes uncoupled and this reduces the viability of NO and increases oxidative stress. This results in endothelial cell dysfunction with increased arginase enzyme activity and damage to the diabetic kidney. Therefore, we conclude that the substrate for L-arginine that maintained glomerular filtration rate in our diabetic model was eNOS and not arginase enzyme since we observed a beneficial effect and not a detrimental effect on renal function with L-arginine supplementation [18, 19].

Relative nitrotyrosine observations resulted in lower levels in the plasma for LA supplemented T2DM rats when compared to the diabetic control rats [20]. This is a positive finding in terms of the NO biosynthetic pathway because it means that there are higher levels of nitrotyrosine in the urine. Based on the NO pathway, cell damage via oxidation and chlorination would be bypassed (Figure 1). Nitrite levels remained similar between all groups, with no significant differences. This is to be expected due to auto oxidation in the NO pathway and presumably equal concentrations of nitrite that remained in the plasma and promoted cell protection. This could be further investigated by testing the plasma nitrite levels. Nitrate levels should be tested to confirm contributions from iNOS and the NO pathway. If nitrate concentrations in the urine are high, then peroxynitrite, from iNOS, has been inactivated and cell damage or death is avoided (Figure 1) [21].

There were no apparent differences among the groups in reference to body weight and fasting glucose. These findings are important because these results clearly show that alterations in the NO biosynthetic pathway precede the observance of abnormally high glucose levels and higher body weights at the beginning of the study. However, the Diabetic Control group did increase in body weight much more rapidly than the Nondiabetic Controls as the study progressed into week 3 and beyond. The GTT shows that LA supplementation does improve glucose tolerance in diabetic rats compared to the Diabetic Controls. Blouet et al. [4] also showed improvement in glucose tolerance and insulin sensitivity in T2DM rats and reported that one of the possible mechanisms was alterations in the biosynthetic NO pathway.

Together, these findings imply that LA supplementation has beneficial effects in preserving renal function in T2DM subjects when implemented at the initial time of diagnosis. In future studies, alterations in the NO pathway need to be determined. To further prove and solidify these study's findings, the plasma and urinary nitrate, plasma cGMP, urinary nitrite, and urinary nitrotyrosine levels should be tested. Also, the different stages of diabetes and an extended experimental timeline should be considered. For example, future studies may include focusing on the effects of LA supplementation on calcium channels in the vasculature, since Awumey et al. determined that eNOS and NO production regulate extracellular calcium-induced relaxation [22]. Observing LA supplementation *in vivo* to investigate the renal microvascular vasodilatory effects to NO-mediated vasodilators would provide further insight into the mechanism of action of LA nutrient supplementation. The inexpensive dietary LA supplementation could be considered a nutritional supplement administered to T2DM patients, as an alternative to other available antidiabetic drugs.

## 5. Conclusions

L-arginine may be an inexpensive alternative treatment for type 2 diabetics. In this study, early intervention with L-arginine supplementation was beneficial by preserving glomerular filtration rates, presumably via increased renal endothelial nitric oxide synthase levels leading to renal vasodilation; however, additional studies are needed to examine the alterations in the other many mediators involved in the nitric oxide pathway to further support this claim. Lastly, there was an improvement in the insulin sensitivity in the LA treated diabetic rats versus versus untreated diabetic rats.

## Figures and Tables

**Table 1:** Effect of high sucrose diet and L-Arginine supplementation on eNOS/*β*-actin ratio in kidney cortex and medulla.

	Control		High sucrose diet		+L-arginine supplement	
	Cortex	Medulla	Cortex	Medulla	Cortex	Medulla
*N*	8	7	8	7	7	7
eNOS monomers/*β*-actin	2.297 ± 0.617	2.972 ± 0.564	2.472 ± 0.176	2.356 ± 0.352	6.622 ± 2.876	15.961 ± 7.043^1,2^
eNOS dimers/*β*-actin	0.656 ± 0.180	1.620 ± 0.274*	0.065 ± 0.045^1^	0.023 ± 0.023^1^	0.138 ± 0.090^1^	0.205 ± 0.205^1^
eNOS total/*β*-actin	2.953 ± 0.777	4.593 ± 0.825	2.537 ± 0.189	2.379 ± 0.357	6.759 ± 2.965	16.167 ± 7.170^1,2^
Dimer/monomer ratio	0.348 ± 0.057	0.599 ± 0.071*	0.025 ± 0.017^1^	0.008 ± 0.008^1^	0.008 ± 0.005^1^	0.005 ± 0.005^1^

*P < 0.05 versus cortex, ^1^ *P* < 0.05 versus control, and ^2^ *P* < 0.05 versus high sucrose diet.

**Table 2:** Effect of a high sucrose diet and L-arginine supplementation on iNOS/*β*-actin ratio in the kidney cortex and medulla.

	Control		High sucrose diet		+L-arginine supplement	
	Cortex	Medulla	Cortex	Medulla	Cortex	Medulla
*N*	8	7	7	6	9	9
iNOS monomers/*β*-actin	3.432 ± 0.511	3.908 ± 0.928	2.568 ± 0.165	2.383 ± 0.174^1^	2.383 ± 0.114	2.292 ± 0.225^1^
iNOS dimers/*β*-actin	1.146 ± 0.223	3.244 ± 0.667*	0.105 ± 0.071^1^	0.419 ± 0.262^1^	0.011 ± 0.007^1^	0.042 ± 0.040^1^
iNOS total/*β*-actin	4.578 ± 0.654	7.151 ± 1.540*	2.673 ± 0.221	2.802 ± 0.396^1^	2.395 ± 0.118^1^	2.333 ± 0.235^1^
Dimer/monomer ratio	0.352 ± 0.063	0.983 ± 0.157*	0.035 ± 0.024^1^	0.154 ± 0.097^1^	0.004 ± 0.003^1^	0.016 ± 0.016^1^

*P < 0.05 versus cortex, ^1^ *P* < 0.05 versus control, and ^2^ *P* < 0.05 versus high sucrose diet.
